# P02.117. Genomic expression changes underlying mind-body practices

**DOI:** 10.1186/1472-6882-12-S1-P173

**Published:** 2012-06-12

**Authors:** J Denninger, J Dusek, M Bhasin, J Huffman, L Slipp, M Scult, B Mahoney, B Chang, R Zusman, H Benson, T Libermann, G Fricchione

**Affiliations:** 1 Benson-Henry Institute at Massachusetts General Hospital, Boston, USA; 2 Institute for Health and Healing ,Abbott Northwestern Hosp., Minneapolis, USA; 3 Beth Israel Deaconess Medical Center, Boston, USA; 4 Psychiatry, Massachusetts General Hospital, Boston, USA; 5 Cardiology, Massachusetts General Hospital, Boston, USA; 6 Boston University School of Public Health, Boston, USA

## Purpose

Genomic markers that reflect responsiveness to a relaxation response (RR) intervention have been detected in healthy volunteers, but have not been examined in clinical conditions for which RR is effective (e.g., hypertension). This study aimed to explore the impact of an RR intervention on gene expression in hypertensive individuals.

## Methods

We performed transcriptional profiling analyses on the Peripheral Blood Mononuclear Cells (PBMCs) obtained before (Pre RR) and after (Post RR) 8 weeks of intervention from a pilot group of 4 hypertensives. Transcriptional profiling used Affymetrix HT HG-U133+ PM arrays, containing >47,000 transcripts corresponding to 38,500 genes. The arrays were normalized using the robust multi-chip analysis (RMA) algorithm. After normalization and preprocessing, differentially expressed genes were identified using the random variance model based t-test. To understand the underlying biological mechanisms associated with RR regulated genes, we performed Gene ontology (GO), pathways and geneset enrichment analysis (GSEA).

## Results

A total of 474 transcripts were significantly differentially expressed (p<0.05) between the Pre and Post RR conditions. Hierarchical clustering of the top differentially expressed genes demonstrates striking segregation between Pre and Post RR conditions. The GO analysis identified significantly affected categories (p<0.05) that include “mRNA metabolic process,” ”positive regulation of RNA metabolic process,” “Calcium signaling pathway” and “programmed cell death.” The pathway analysis identified the over-representation (p<0.05) of differentially expressed genes to “cell cycle G/M checkpoint regulation,” “P53 signaling,” “Inositol metabolism signaling,” “Apoptosis Signaling,” “MIF Regulation of Innate Immunity,” and “Cardiac β-adrenergic Signaling.” Furthermore, GSEA analysis identified upregulation of 263 genesets (p <0.05) in Post RR including phosphatidylinositol signaling system, IFN-γ endothelial up, B cell receptor signaling pathway, FAS pathway, VEGF signaling pathway, Cardiac EGF pathway and MAPK pathway. Additional results to follow.

## Conclusion

These results suggest specific biochemical pathways on which to expand future research in the study of mind/body therapies in clinical populations.

